# APOE ɛ4 Is Associated with Postprandial Inflammation in Older Adults with Metabolic Syndrome Traits

**DOI:** 10.3390/nu13113924

**Published:** 2021-11-02

**Authors:** Yannik Bernd Schönknecht, Silke Crommen, Birgit Stoffel-Wagner, Martin Coenen, Rolf Fimmers, Peter Stehle, Alfredo Ramirez, Sarah Egert

**Affiliations:** 1 Nutritional Physiology, Department of Nutrition and Food Science, University of Bonn, 53115 Bonn, Germany; yschoenknecht@uni-bonn.de (Y.B.S.); s.crommen@uni-bonn.de (S.C.); p.stehle@uni-bonn.de (P.S.); 2 Institute of Clinical Chemistry and Clinical Pharmacology, University Hospital Bonn, 53127 Bonn, Germany; birgit.stoffel-wagner@ukbonn.de; 3 Clinical Study Core Unit, Study Center Bonn, University Hospital Bonn, 53127 Bonn, Germany; martin.coenen@ukbonn.de; 4 Institute of Medical Biometry, Informatics and Epidemiology, University Hospital Bonn, 53127 Bonn, Germany; fimmers@imbie.meb.uni-bonn.de; 5 Division of Neurogenetics and Molecular Psychiatry, Department of Psychiatry and Psychotherapy, University Hospital Cologne, 50931 Cologne, Germany; alfredo.ramirez@uk-koeln.de; 6 Department of Neurodegenerative Diseases and Geriatric Psychiatry, University Hospital Bonn, 53127 Bonn, Germany; 7 German Center for Neurodegenerative Diseases (DZNE), 53127 Bonn, Germany; 8 Department of Psychiatry and Glenn Biggs Institute for Alzheimer’s and Neurodegenerative Diseases, San Antonio, TX 78229, USA; 9 Excellence Cluster on Cellular Stress Responses in Aging-Associated Diseases (CECAD), University of Cologne, 50931 Cologne, Germany

**Keywords:** apolipoprotein E gene polymorphism, dietary pattern, inflammation, oxidative stress, postprandial state

## Abstract

The apolipoprotein E (*APOE*) polymorphism impacts blood lipids and biomarkers of oxidation and inflammation, contributing to an isoform-dependent disease risk. We investigated the effect of the *APOE* genotype on postprandial metabolism after consumption of three different isoenergetic (4200 kJ) meals in older adults with a CVD risk phenotype. In a randomized crossover study, participants with metabolic syndrome traits (*APOE* E3, n = 39; E4, n = 10; mean age, 70 ± 5 years; BMI 31.3 ± 3.0 kg/m^2^) consumed a Western-like diet high-fat (WDHF), Western-like diet high-carbohydrate (WDHC), or Mediterranean-like diet (MED) meal. Parameters of lipid and glucose metabolism, inflammatory, and oxidative parameters were analyzed in blood samples collected at fasting and 1–5 h postprandially. Data were analyzed by linear mixed models. The magnitude of the IL-6 increase after the WDHF meal was significantly higher in E4 than in E3 carriers (iAUC: E4 = 7.76 vs. E3 = 2.81 pg/mL × h). The time to detect the IL-6 increase was shorter in the E4 group. All meals produced postprandial glycemia, insulinemia, and lipidemia, without differences between the E3 and the E4 groups. IL-1β and oxidized LDL levels did not change postprandially. In conclusion, APOE E4 carriers display increased postprandial inflammation, indicated by higher postprandial IL-6 increase, when compared to non-carriers.

## 1. Introduction

The apolipoprotein E (APOE) genotype is an established risk factor for cardiovascular disease (CVD), neuropathology, and Alzheimer’s disease [1,2,3]. In humans, apoE is expressed as three different isoforms (E2, E3, and E4) that arise from three different alleles (ε2, ε3, and ε4, respectively), which are present with in varying frequency of 5–10% ε2, 65–70% for ε3, and 15–20% for ε4. These alleles give rise to three homozygous (APOE2/E2, APOE3/E3, and APOE4/E4) and three heterozygous (APOE3/E2, APOE4/E3, and APOE4/E2) genotypes [2]. The APOE4 carrier status is predictive of CVD and Alzheimer’s disease [1,2,3]. Although the pathological associations of APOE4 have been investigated widely, the main etiological mechanisms have not been clearly defined.

The protein apoE is involved in multiple biological pathways and processes, including lipoprotein metabolism and intracellular cholesterol utilization [4,5], immunoregulation [6], (neuro)inflammation [4], and neuroprotection [7]. In addition, the APOE genotype appears to be an important determinant of individual responsiveness to dietary factors [2,8]. For example, beneficial effects of the flavonol quercetin on arterial blood pressure (BP) [9] have been observed in individuals carrying the ε3 allele, but not in those carrying the ε4 allele [2]. By contrast, Griffin et al. recently found that the E4 genotype is associated with lower plasma cholesterol and apoB levels than the wild-type genotype (E3/E3) after replacement of dietary saturated fatty acids (SFAs) with low glycemic index carbohydrates [10]. In addition, the APOE genotype may predict the lipid and lipoprotein responses to marine n-3 fatty acid interventions [11,12,13]. Carriers of the ɛ4 allele are low responders to docosahexaenoic acid supplements, suggesting that they may need higher intakes of docosahexaenoic acid for cardiovascular or other health benefits than non-carriers [14].

Most studies investigating the interactive effects of the APOE genotype and dietary factors on cardiometabolic parameters have been performed in the fasted state. However, there is growing evidence that postprandial metabolism contributes to CVD [15,16], which is particularly relevant given that most individuals are for most of the day in the fed state. Postprandial metabolism is characterized by metabolic (lipidemia, glycemia/insulinemia), oxidative, and immune imbalances; this phenomenon is termed “postprandial oxidative stress” and is accompanied by low-grade inflammation and impaired endothelial function [17,18,19]. Magnitude and duration of postprandial responses are highly variable and are influenced by dietary, physiological, and genetic factors. Previous studies reported effects of the APOE genotype on postprandial lipemia. The presence of the ɛ4 allele is associated with increased postprandial triglyceride responses after a fat overload compared to the E3/E3 genotype [20,21]. However, current knowledge of the interactive effects of the APOE genotype and meals representing different dietary patterns on postprandial lipid, glucose, and inflammatory outcomes is extremely limited.

The present secondary analysis explored the interactive effects of the APOE genotype (E4 vs. E3) and acute intake of isoenergetic meals representing Mediterranean and Western diets on postprandial lipemic, glycemic, and inflammatory responses. Data were obtained from the Diet–Body–Brain (DietBB) postprandial study, which was conducted in 60 older individuals with a cluster of metabolic syndrome traits [22]. We hypothesized that the APOE genotype would influence metabolic syndrome risk markers, both at baseline and in response to intake of the different meals. The participants were retrospectively genotyped and data were re-analyzed. The overall effects of the test meals evaluated in the study are not the focus of the current study, as these data have been reported previously [22].

## 2. Materials and Methods

### 2.1. Participants

**Figure 1:**
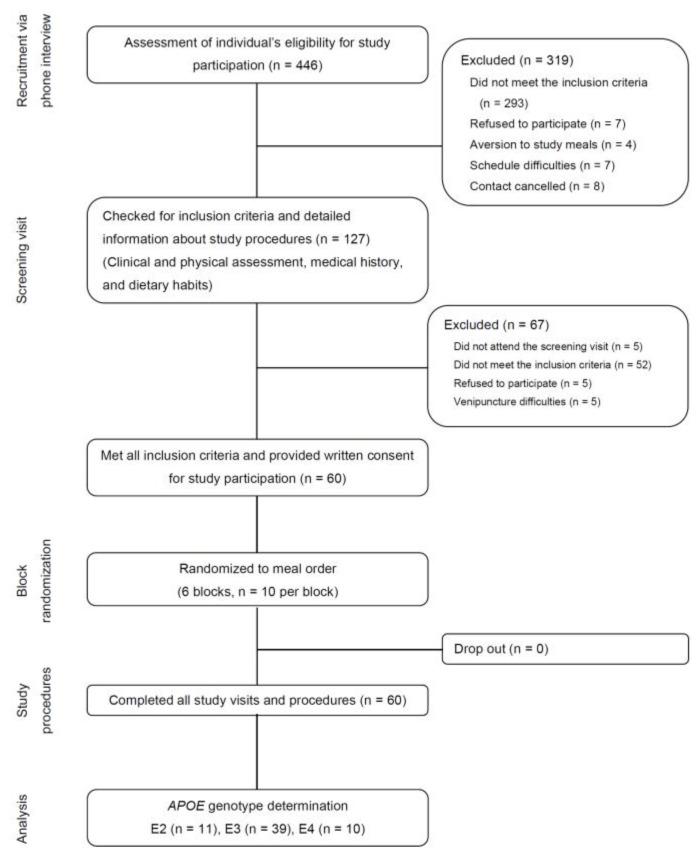
Flow diagram of participant inclusion/exclusion (adapted from 22).

Full details of the study design and participant recruitment, enrolment, and randomization have been described previously (Figure 1) [22]. Of the 446 individuals who expressed an interest in the study, 127 individuals aged 60–80 years with a BMI > 27 kg/m^2^ attended the initial screening, which included physical assessments (body height and weight, resting BP, heart rate, and waist and hip circumference), laboratory assessments (serum creatinine, urea, sodium, potassium, bilirubin, uric acid, gamma-glutamyl transferase, alanine transaminase, aspartate transaminase, hepatic lipase, blood counts, lipids and lipoproteins, glucose, insulin, HbA1c, and high-sensitivity C-reactive protein (hs-CRP)), medical history, and a dietary questionnaire. Participants were included if they displayed at least three out of the following traits of metabolic syndrome: (i) visceral fat distribution (waist circumference ≥ 94 cm for men and ≥80 cm for women), (ii) pre-hypertension (systolic BP ≥ 120–139 mmHg and/or diastolic BP ≥ 80–89 mmHg) or hypertension (systolic BP ≥ 140–159 mmHg and/or diastolic BP ≥ 90–99 mmHg), (iii) elevated fasting glucose (≥5.55 mmol/L), dyslipidemia (fasting serum triglycerides ≥ 1.7 mmol/L or serum HDL cholesterol < 1.0 mmol/L for men and <1.3 mmol/L for women), and/or a pro-inflammatory state (high-sensitivity C-reactive protein ≥ 2.0 mg/dL). The following were defined as main criteria: smoking, insulin-treated diabetes mellitus, long-term dietary supplements intake, inflammatory disease, disease of the liver, kidney, or gastrointestinal tract, a history of cardiovascular events, abnormal thyroid function, cancer, recent major surgery or illness, substance or alcohol abuse, participation in a weight loss program, and malabsorption syndromes [22].

The study protocol was explained in detail to all participants, and written informed consent was obtained prior to the study. All study procedures were approved by the Ethics Committee of the Medical Faculty of the Rheinische Friedrich-Wilhelms-Universität Bonn, Germany. The study was conducted in accordance with the guidelines of the Declaration of Helsinki. The trial was registered at www.germanctr.de/ and http://apps.who.int/trialsearch/ as DRKS00009861 (accesseded on 22 January 2016).

The participants were instructed to maintain their usual diet, level of physical activity, lifestyle, and body weight throughout the study period. Participants requiring antihypertensive agents, lipid-lowering drugs, metformin, or thyroid therapy were instructed to continue their usual regimen throughout the study. Participants were instructed to complete a 3-day food diary prior to each study visit, in order to monitor and identify potential variations in total energy and macronutrient intakes [22].

### 2.2. Study Protocol

This study was a randomized, dietary-controlled crossover trial. Each individual participated in three 5-h meal tests. Participants were assigned to the three different test meals by a block randomization method, as described previously [22].

Participants were advised to not consume alcohol on the day before the test and to avoid intensive physical activity for 12 h prior to the test. In addition, participants were advised to standardize their meal intake on the evening before the test. The tests were then conducted in the morning after an overnight fast (≥10 h). Venous blood sampling was conducted prior to (0 h) and 1, 2, 3, 4, and 5 h after consuming the test meal.

Participants consumed an isoenergetic (4200 kJ per meal) and isonitrogenous test meal at each visit. The test meals were designed to represent real-life conditions and included a Western typical diet high-fat (WDHF) meal, a Western typical diet high-carbohydrate meal (WDHC), and a Mediterranean typical diet (MED) meal. The nutrient compositions of the test meals (Table 1) were calculated using the computer-based nutrient calculation program EBISpro (University of Hohenheim, Stuttgart, Germany) and the German Nutrient Database Bundeslebensmittelschlüssel, version 3.01 (Max-Rubner-Institut, Karlsruhe, Germany). Details of the meal preparation and food components have been reported previously [22]. All participants completed the test meal within 20 min under observation by a member of the laboratory staff.

### 2.3. Measurments

#### 2.3.1. Anthropometrics, Body Composition, and Blood Pressure

Body height and weight were determined to the nearest 0.1 cm and 0.1 kg, respectively, using a scale with an integrated stadiometer. Waist and hip circumference and body composition measurements were conducted as previously reported [22]. BP and heart rate were measured with an automatic BP measurement device (Boso Carat Professional) under standardized conditions, according to the recommendations of the American Heart Association [23].

#### 2.3.2. Blood Sample Processing

Details of the pre-analytical procedures of fasting and postprandial blood collection were described previously Whole blood, plasma, and serum aliquots were frozen in cryovials and stored at −80 °C until analysis. All analyses were performed in a blind manner [22]. 

#### 2.3.3. APOE Genotype Determination

Genomic DNA was isolated from EDTA blood using standard procedures. Assignment of APOE allele status was performed by genotyping the two single nucleotide polymorphisms defining ε2, ε3, and ε4 in APOE, i.e., rs429358 and rs7412. To this end, a TaqMan allelic discrimination assay (Thermo Fisher Scientific) was used on a StepOnePlus™ Real-Time PCR system, following the manufacturer’s instructions (Thermo Fischer Scientific, Waltham, MA, USA). Positive controls were included to ensure accuracy of the assay. Ambiguous results were re-analyzed.

#### 2.3.4. Serum Lipids, Non-Esterified Fatty Acids, Insulin, and Plasma Glucose

The serum total cholesterol concentration was determined via polychromatic endpoint measurement, whereas serum concentrations of HDL cholesterol, LDL cholesterol, and triglycerides were assessed via endpoint measurement with a Dimension Vista 1500 analyzer (Siemens Healthcare Diagnostics, Erlangen, Germany). Concentrations of non-esterified fatty acids (NEFAs) in serum were determined using a commercially available colorimetric enzyme assay (Wako Chemicals GmbH, Neuss, Germany) [22]. The plasma glucose concentration was assessed via bichromatic endpoint measurement with a Dimension Vista 1500 analyzer. Serum insulin concentrations were determined using a chemiluminescent-immunometric assay with an Immulite 2000 analyzer (Siemens Healthcare Diagnostics, Erlangen, Germany).

#### 2.3.5. Biomarkers of Inflammation and Endothelial Activation

The fasting and postprandial levels of IL-1β (high sensitivity, hs) and hs-IL-6, and the soluble adhesion molecules E-selectin (sE-selectin), intercellular adhesion molecule-1 (sICAM-1), and vascular cell adhesion molecule-1 (sVCAM-1), were analyzed in duplicate using commercially available enzyme-linked immunoassay kits (IL-1β, IL-6, sE-selectin, sICAM-1, sVCAM-1; R & D Systems) [22]. Serum hs-CRP was determined using a turbidimetric immunoassay (Cobas 8000 modular analyzer series).

#### 2.3.6. Biomarkers of Oxidation and the Antioxidant State

Plasma oxidized LDL (oxLDL) levels were determined in duplicate using commercially available enzyme-linked immunoassay kits (Immundiagnostik). Plasma concentrations of vitamin C, α-tocopherol, retinol, and β-carotene were determined via high-performance liquid chromatography, as described previously [22]. The total antioxidant capacity of plasma samples was assessed by the Trolox equivalent capacity (TEAC) method [24].

### 2.4. Statistical Analyses

The Statistical analyses were conducted using the SPSS statistical software package (version 23; IBM, Armonk, NY, USA). To detect potential differences in total energy and macronutrient intake on the days prior to testing (as recorded in the 3-day food diaries), the data were compared using a one-way ANOVA. Genotype-related differences between the baseline characteristics of the participants at screening were analyzed using unpaired Student’s *t*-tests. Prior to analyses, glucose, insulin, and hs-CRP data were log-transformed.

Linear mixed models were used to test for the effects of genotype, time, and test meal on postprandial outcomes. Fasting values were used as covariates, and participants were used as random factors. When there was no evidence of interactions (*p* > 0.05), mixed model calculations were repeated without the interaction terms. When there was evidence for significant interactive effects, the data were analyzed by performing linear mixed model calculations as post-hoc analyses. Differences between the fasting variables were analyzed using a mixed model analysis. Prior to analyses, glucose, insulin, IL-6, sICAM-1, sVCAM-1, TEAC, and β-carotene data were log-transformed. Residuals obtained from the calculations were inspected for normality to control for the fit of the statistical model. Logarithmic transformation was applied before analysis if the residuals were not normally distributed.

Summary measures for postprandial responses were calculated as the area under the curve (AUC). The incremental AUC (iAUC) was calculated to determine the concentrations of all postprandial study variables. Calculations were performed as described previously [25]. Statistical analyses of the iAUC data were performed using a linear mixed model, including test meal and APOE genotype as fixed factors and participants as random factors. Data are expressed as the mean ± SD or SEM, and all *p*-values listed are two-sided.

## 3. Results

### 3.1. APOE Genotype Distribution and Baseline Characteristics

Of the 60 study participants, 39 were homozygous for the ε3 allele (ε3/ε3 genotype), 11 carried the ε2/ε3 genotype, one carried the ε2/ε4 genotype, and nine carried the ε3/ε4 genotype. We did not observe participants homozygous for the ε2 or ε4 allele. The participants were classified into three groups: (i) E2 (n = 11), carrying the ε2/ε3 genotype; (ii) E3 (n = 39), carrying the ε3/ε3 genotype; and (iii) E4 (n = 10), carrying the ε2/ε4 or ε3/ε4 genotype. The E2 group was excluded from further statistical analyses. 

Age, body mass, BMI, waist circumference, waist-to-height ratio, fat mass, systolic and diastolic BP, pulse rate, plasma glucose, and serum insulin, triglyceride, total cholesterol, LDL cholesterol, HDL cholesterol, and CRP levels were comparable between the E3 and E4 groups (Table 2). Furthermore, there were no significant differences in energy and nutrient consumption between the APOE groups (data not shown), and the diet diaries indicated that the habitual diets of the participants remained unchanged throughout the study (data not shown) [22].

### 3.2. Serum Triglycerides, NEFAs, Insulin, and Plasma Glucose

The APOE genotype did not influence the postprandial triglyceride or NEFA response (Table 3). The glucose iAUC was higher for the WDHC group than for the WDHF and MED groups (*p* = 0.006), although there was no effect of the APOE genotype. Similarly, although there was a significant effect of the test meal (*p* < 0.001), statistical analyses of the insulin iAUC did not detect an APOE genotype effect.

### 3.3. Plasma IL-6, IL-1β, and Endothelial Adhesion Molecules

**Figure 2:**
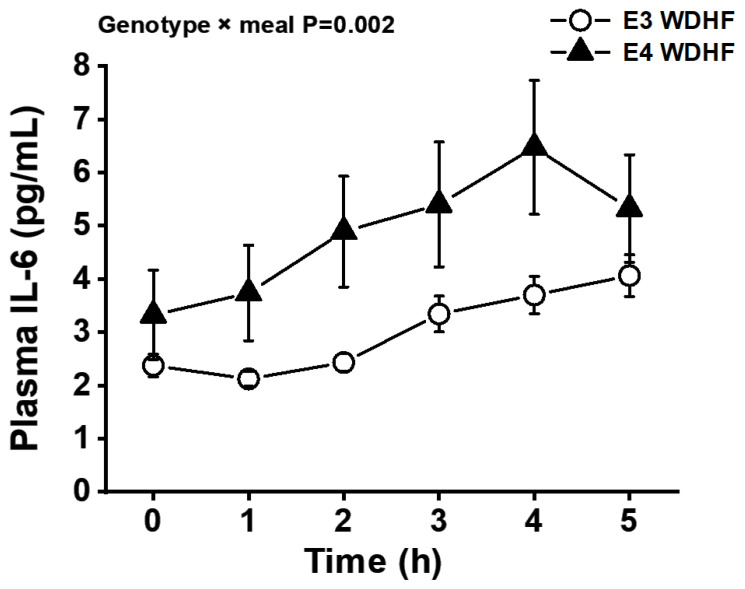
Effect of the APOE genotype (E3 vs. E4) on the plasma IL-6 response to a Western-like diet high-fat (WDHF) meal in high CVD risk individuals. Data are represented as the mean ± SEM. *p* = 0.002 for genotype × test meal interaction; *p* < 0.001 for the fixed factor time.

**Figure 3:**
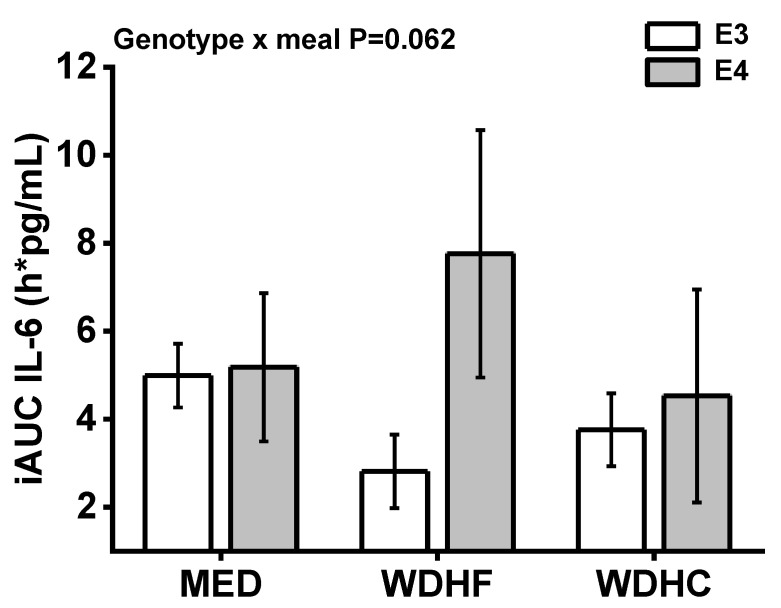
Effects of the APOE genotype (E3 vs. E4) on postprandial changes in plasma IL-6 levels in high CVD risk individuals after the consumption of three different meals, as indicated by incremental AUC (iAUC) values. Data are represented as the mean ± SEM. Genotype × meal interaction, *p* = 0.062. MED, Mediterranean-like diet meal; WDHC, Western-like diet high-carbohydrate meal; WDHF, Western-like diet high-fat meal.

Postprandial IL-6 concentrations were significantly higher than the fasting (pre-meal) levels in all test meal groups. Statistical analyses detected a significant APOE genotype × time interaction (*p* = 0.008), with E4 participants displaying higher IL-6 concentrations than E3 participants at the 1–2 h postprandial time-points. Furthermore, a significant APOE genotype × meal interaction (*p* = 0.002) was detected, with E4 participants displaying a larger IL-6 response to the WDHF meal than E3 participants (Figure 2). Similarly, the IL-6 AUC values showed a statistical trend for a APOE genotype × meal interaction (*p* = 0.062) (Table 3, Figure 3). The plasma IL-1β level showed neither postprandial changes nor any influence of APOE genotype. In addition, the APOE genotype had no effect on the AUC values, fasting concentrations, or postprandial changes in the levels of sE-selectin, sICAM-1, or sVCAM-1 (Table 3).

### 3.4. Plasma TEAC and oxLDL, Vitamin C, Retinol, α-Tocopherol, and β-Carotene

In the fasting state, plasma oxLDL concentrations in the E4 group were significantly higher than those in the E3 group (E4, 168.4 ± 124.1 ng/mL; E3, 89.4 ± 43.8 ng/mL; *p* = 0.002; mean ± SD). However, the oxLDL concentration was not affected by meal intake, and the oxLDL iAUC did not differ between the E3 and E4 genotype groups (Table 3). The APOE genotype had no significant effects on AUC values, fasting concentrations, or postprandial changes in plasma TEAC or levels of vitamin C, α-tocopherol, β-carotene, or retinol (Table 3).

## 4. Discussion

In view of the fact that the postprandial response is a significant contributor to CVD risk, this study examined for the first time the impact of the APOE genotype (E4 vs. E3) on the postprandial responses of older adults with metabolic syndrome traits to three differently composed meals. The major finding was that the APOE genotype influences the postprandial IL-6 response, with carriers of the ε4 allele (E4 group) displaying markedly higher plasma IL-6 concentrations than ε3/ε3 individuals (E3 group) after the consumption of a WDHF meal. We did not find an effect of apoE genotype on the IL-6 response to MED and WDHC meal. In addition, fasting plasma oxLDL concentrations in the E4 group were significantly higher than those in the E3 group. By contrast, we did not detect an effect of the APOE genotype on the time course and the iAUC of postprandial triglycerides, NEFAs, glucose, insulin, or parameters of endothelial activation and oxidation. 

In chronic diseases, such as metabolic, vascular, and neurodegenerative diseases, low-grade inflammation is seen as an important underlying disease mechanism, wherein poor diet (e.g., regular intake of high-energy, high-fat meals) likely induces and maintains a pro-inflammatory status [16,17,26,27]. A number of studies in cell lines, targeted replacement rodents, and human volunteers have suggested an association between the APOE ɛ4 allele and a pro-inflammatory state [4,28], which may contribute to increased disease risk. In the present study, the fasting concentration of IL-6, CRP, IL-1β, and markers of endothelial activation did not differ between E3 and E4 carriers. The lack of postprandial changes in IL-1β is consistent with the results of other studies that infrequently show postprandial changes of IL-1β concentrations [17]. However, postprandial inflammation, indicated by the IL-6 response, was higher in the E4 group than in the E3 group. Postprandial inflammation is mediated by redox-sensitive transcription factors, including nuclear factor kappa-light-chain-enhancer of activated B-cells (NF-κB), the main mediator of inflammatory responses [29]. Notably, activation of NF-κB in transgenic E4 mice is more pronounced and prolonged than in E3 transgenic mice [30]. Furthermore, a study using stably transfected murine macrophages expressing human APOE found that E4-expressing macrophages displayed enhanced NF-κB activation, higher levels of pro-inflammatory cytokines, and lower levels of anti-inflammatory IL-10 than E3-expressing macrophages [31]. Thus, we speculate that the increased IL-6 response seen in ɛ4 allele carriers may be the result of enhanced NF-κB activation. Furthermore, oxidative stress is also implicated in the inflammatory response, and baseline oxLDL levels in E4 participants were higher than those in E3 participants, indicating an enhanced oxidative state in ɛ4 carriers, which may contribute to the augmented inflammatory response. In support of this hypothesis, other groups have also reported an association between APOE4 and elevated markers of oxidative stress [28].

In the present study, the APOE genotype did not influence lipemic responses to the three test meals. Previous studies investigating the effects of the APOE genotype on postprandial lipidemia have produced inconsistent findings. For example, in normolipidemic men, Calabuig-Navarro et al. [20] observed little impact of the APOE genotype on the postprandial plasma triglyceride response to a high-fat test meal enriched with unsaturated fatty acids, SFAs, or SFAs with fish oil. By contrast, Cardona et al. [21] found that metabolic syndrome patients with a genotype other than E3/E3 had a greater risk for postprandial hypertriglyceridemia after a fat overload than E3/E3 individuals. In addition, greater and longer-lasting postprandial hypertriglyceridemia has been reported in patients with familial combined hyperlipidemia who have the ɛ4 allele [32]. These results suggest that the effect of the APOE genotype on postprandial triglyceride responses may be greater in hyperlipidemic individuals than in normolipidemic individuals. In the DISRUPT cohort, E4 carriers had higher fasting triglyceride levels than wild-type E3/E3 individuals, resulting in a 22% increase in the triglyceride AUC [33]. Subdivision of the E4 carriers according to age (≤50 years and >50 years) revealed that the effect was only evident in the >50 years age group. These results contradict our current findings, since we did not find a significant effect of the APOE genotype on serum triglyceride levels in the 60–80-year-old participants (Table 2). These inconsistent findings suggest that the impact of the APOE genotype on the postprandial triglyceride response may depend on other key physiological variables. 

We found no significant effects of the APOE genotype on postprandial glucose, insulin, and NEFA responses. Currently, only limited data are available regarding the impact of the APOE genotype on these outcomes and the potential underlying mechanisms. Nonetheless, our findings are consistent with those of Calabuig-Navarro [20], who did not find a significant meal × genotype interaction for the summary measures of glucose, insulin, and NEFA. In addition, Jackson et al. [34] did not observe an effect of the APOE genotype on NEFA and glucose responses in normolipidemic men, but did report a genotype effect for the postprandial insulin response (*p* ≤ 0.012), with a tendency for lower insulin responses after meal consumption in the heterozygous APOE3/E4 group [34]. 

To the best of our knowledge, the current study is the first to report that the level of postprandial inflammation after a meal typical of a Western diet is influenced by the APOE genotype, with a stronger inflammatory response in E4 carriers than in E3 homozygotes. One of the strengths of our study is that we focused on a clinically relevant population, overweight/obese older individuals, and used a whole diet approach to study the acute effects of meals on a wide array of cardiometabolic parameters. By contrast, trials that apply isolated nutrient (e.g., high-fat) challenges are unlikely to identify the entire range of metabolic stimuli [22]. However, our study has some limitations. It was originally designed to examine the effects of three different dietary patterns on postprandial metabolism in older adults with a high CVD risk phenotype [22]. In view of the contribution of apoE to lipoprotein metabolism, oxidation, inflammation, and endothelial function, and the effects of the APOE genotype on fasting lipids and responsiveness to dietary manipulation, we decided to reanalyze our results in relation to allele variants of this gene. We hypothesized that the secondary analysis would reveal potential APOE genotype-specific responses to meal intake not considered previously because data were not analyzed in terms of APOE gene polymorphisms. Retrospective genotyping of the 60 participants resulted in a relatively small and unbalanced distribution throughout the APOE subgroups (especially ɛ4 and ɛ2 allele carriers), which reduced the power of the study to detect differences in the postprandial responses to meal intake. In addition, it is possible that the effect of the APOE allele is dependent on heterozygosity (APOE ε3/ε4) and homozygosity (APOE ε4/ε4). Thus, future postprandial studies should consider prospective APOE genotyping during screening analysis to enable the recruitment of equal numbers of APOE ε3/ε3, ε3/ε4, and ε4/ε4 participants. This approach would facilitate the determination of the effects of meal composition on postprandial metabolism in relation to the APOE genotype. Moreover, consensus is missing on the optimal marker for postprandial inflammation assessment [17]. In future studies, other parameters besides IL-6, such as NF-κB activation and transcription factors that are activated by cytokines, may be included in the assessment to investigate the influence of APOE on postprandial inflammation.

In conclusion, secondary analysis of the study data according to APOE genotype revealed that overweight/obese E4 carriers display increased postprandial inflammation, indicated by higher postprandial IL-6 increase, when compared to non-carriers. This effect may contribute to the enhanced CVD risk of E4 carriers compared to non-carriers. However, our results are based on a relatively small number of ɛ4 allele carriers in a retrospectively genotyped cohort and should be confirmed in a prospectively genotyped cohort. Nevertheless, the data presented here indicate that the APOE genotype may be an important determinant of the postprandial response in dietary intervention studies.

## Figures and Tables

**Table 1:** Energy contents and nutrient compositions of the test meals (adapted from 22).

Energy/Nutrient	MED Meal	WDHF Meal	WDHC Meal
Energy, kJ	4238	4230	4241
Energy density, kJ/g	5.6	7.2	9.8
Carbohydrates, g	133	94	145
Carbohydrates, EN %	53	37	58
Mono- and disaccharides, g	51	45	87
Polysaccharides, g	79	47	57
Ratio of polysaccharides to mono- and disaccharides	0.6	1.0	1.5
Dietary fiber, g	14	4	5
Protein, g	26	26	26
Protein, EN %	10	10	10
Total fat, g	40	59	34
Total fat, EN %	36	53	31
SFA, g	6	32	19
MUFA, g	24	20	11
PUFA, g	9	4	2
β-carotene, mg	4.8	0.2	2.3
Retinol, mg RE	832	365	522
Vitamin E, mg TE	10.8	2.3	2.9
Vitamin C, mg	102	9	16

EN %, energy percent; MED, Mediterranean-like diet meal; mg RE, mg of retinol equivalent; mg TE, mg of α-tocopherol equivalent; SFA, saturated fatty acids; WDHC, Western-like diet high-carbohydrate meal; WDHF, Western-like diet high-fat meal.

**Table 2:** Baseline characteristics of the participants according to the *APOE* genotype ^1−3^.

	All	E3	E4	*p*-Value E3 vs. E4 ^4^
n	49	39	10	
Age (years)	69.8 ± 5.4	69.7 ± 5.4	70.4 ± 5.8	0.715
Body weight (kg)	89.7 ± 10.6	89.9 ± 10.7	88.8 ± 10.7	0.773
BMI (kg/m^2^)	31.3 ± 3.0	31.3 ± 3.1	31.2 ± 3.1	0.915
Waist circumference (cm)	106.9 ± 8.2	106.4 ± 7.6	108.7 ± 10.4	0.534
Waist-to-height ratio	0.63 ± 0.05	0.63 ± 0.05	0.64 ± 0.05	0.371
Fat mass (%)	33.9 ± 7.2	34.2 ± 7.3	32.6 ± 7.6	0.558
Systolic BP (mmHg)	148 ± 16	149 ± 17.9	145.5 ± 14.7	0.599
Diastolic BP (mmHg)	88.0 ± 9.3	87.9 ± 9.5	88.7 ± 9.1	0.820
Pulse (min^−1^)	64 ± 11	64 ± 11	64 ± 13	0.881
Plasma glucose (mmol/L)	5.61 ± 1.00	5.71 ± 1.06	5.22 ± 0.60	0.154
Serum insulin (pmol/L)	88.2 ± 35.0	88.4 ± 36.2	87.4 ± 31.6	0.992
Serum triglycerides (mmol/L)	1.87 ± 0.85	1.90 ± 0.85	1.79 ± 0.88	0.739
Serum total cholesterol (mmol/L)	5.28 ± 0.92	5.27 ± 0.90	5.30 ± 1.02	0.928
Serum HDL cholesterol (mmol/L)	1.40 ± 0.32	1.41 ± 0.34	1.33 ± 0.23	0.498
Serum LDL cholesterol (mmol/L)	3.31 ± 0.81	3.26 ± 0.78	3.50 ± 0.94	0.417
Serum hs-CRP (mg/L)	4.2 ± 7.5	3.9 ± 7.7	5.4 ± 2.1	0.608

^1^ Shown as mean ± SD. ^2^ All parameters were measured in fasting blood samples. ^3^ E3, ε3/ε3 genotype; E4, ε2/ε4 (n = 1) or ε3/ε4 genotype (n = 9). ^4^ Comparisons were made using unpaired Student’s *t*-tests. BP, blood pressure; hs-CRP, high-sensitivity C-reactive protein.

**Table 3:** Postprandial responses to three different test meals in high CVD risk participants classified by APOE genotype (E3 vs. E4), as indicated by incremental AUC (iAUC) values for parameters of lipid and glucose metabolism, biomarkers of inflammation, and antioxidant status ^1^.

	E3 (n = 39)			E4 (n = 10)			*p*-Value		
	MED	WDHF	WDHC	MED	WDHF	WDHC	Test Meal	Genotype	Genotype × Meal
Glucose iAUC (mmol/L × h)	4.7 ± 1.1	3.0 ± 0.5	5.1 ± 1.0	3.8 ± 1.4	3.1 ± 0.8	6.8 ± 1.9	0.006	0.810	0.800
Insulin iAUC (pmol/L × h)	1752 ± 187	1489 ± 134	2103 ± 194	2018 ± 498	1380 ± 147	2933 ± 567	<0.001	0.926	0.096
Triglycerides iAUC (mmol/L × h)	3.1 ± 0.3	4.0 ± 0.3	2.9 ± 0.3	2.6 ± 0.5	3.9 ± 0.4	2.9 ± 0.5	<0.001	0.680	0.574
NEFA iAUC (mmol/L × h)	−1.44 ± 0.11	−0.92 ± 0.10	−1.48 ± 0.10	−1.18 ± 0.20	−0.87 ± 0.17	−1.77 ± 0.2	<0.001	0.921	0.161
IL-1β iAUC (pg/mL × h)	0.02 ± 0.02	0.00 ± 0.02	0.03 ± 0.03	−0.03 ± 0.04	0.05 ± 0.04	−0.02 ± 0.04	0.952	0.708	0.270
IL-6 iAUC (pg/mL × h)	4.99 ± 0.72	2.81 ± 0.84	3.76 ± 0.83	5.18 ± 1.69	7.76 ± 2.81	4.53 ± 2.42	0.161	0.289	0.062
sE-selectin iAUC (ng/mL × h)	−3.9 ± 1.2	−4.2 ± 1.4	−3.6 ± 1.9	3.3 ± 5.3	−4.9 ± 2.1	−9.3 ± 2.4	0.728	0.839	0.179
sICAM-1 iAUC (ng/mL × h)	3.3 ± 10.6	−32.7 ± 14.0	−19.5 ± 18.7	−42.5 ± 38.7	−87.6 ± 77.1	−20.7 ± 20.3	0.227	0.090	0.581
sVCAM-1 iAUC (ng/mL × h)	−81.8 ± 40.6	−84.8 ± 46.6	−157.2 ± 90.7	182.8 ± 247.8	−45.8 ± 95.7	−156.1 ± 45.7	0.371	0.277	0.349
oxLDL iAUC (ng/mL × h)	0.9 ± 11.3	14.1 ± 9.5	−1.1 ± 12.8	−23.5 ± 17.3	−0.8 ± 20.9	9.6 ± 27.6	0.474	0.579	0.261
Vitamin C iAUC (mg/L × h)	3.1 ± 1.0	−4.9 ± 0.8	−4.7 ± 1.0	1.4 ± 2.2	−3.1 ± 1.0	−1.3 ± 2.7	<0.001	0.298	0.271
Tocopherol iAUC (µg/mL × h)	−1.8 ± 0.6	−1.3 ± 0.4	−0.5 ± 1.0	−4.3 ± 4.2	−2.0 ± 1.0	−2.2 ± 1.7	0.531	0.279	0.732
β-carotene iAUC (ng/mL × h)	−17.4 ± 13.5	−29.4 ± 14.8	−14.9 ± 26.6	−3.0 ± 33.8	−83.0 ± 30.1	−3.2 ± 36.6	0.334	0.752	0.321
Retinol iAUC (ng/mL × h)	−9.6 ± 22.0	−23.9 ± 19.3	7.8 ± 15.0	−10.6 ± 47.3	−3.7 ± 24.9	8.3 ± 28.2	0.288	0.922	0.988
TEAC iAUC (mmol/L × h)	−0.11 ± −0.05	−0.14 ± 0.005	−0.13 ± 0.05	−0.01 ± 0.09	−0.10 ± 0.18	−0.08 ± 0.12	0.868	0.265	0.921

^1^ Shown as the mean ± SEM. iAUC, incremental area under the curve; MED, Mediterranean-like diet meal; NEFA, non-esterified fatty acid; oxLDL, oxidized low-density lipoprotein; sE-selectin, soluble E-selectin; sICAM-1, soluble intercellular adhesion molecule-1; sVCAM-1, soluble vascular cell adhesion molecule-1; TEAC, Trolox equivalent capacity; WDHC, Western-like diet high-carbohydrate meal; WDHF, Western-like diet high-fat meal.
